# Modeling and simulation of blood flow in unhealthy elliptic arteries with computational fluid dynamics approach

**DOI:** 10.1371/journal.pone.0317989

**Published:** 2025-04-08

**Authors:** Abdul Wahab, Muhammad Imran Asjad, Muhammad Bilal Riaz, Jamil Abbas Haider

**Affiliations:** 1 Department of Mathematics, University of Management and Technology, Lahore, Pakistan; 2 IT4Innovations, VSB – Technical University of Ostrava, Ostrava, Czech Republic; 3 Abdus Salam School of Mathematical Sciences, Government College University Lahore, Lahore, Pakistan; Radiation Application Research School, NSTRI, IRAN, ISLAMIC REPUBLIC OF

## Abstract

This study investigates the influence of varying degrees of stenosis on blood flow within elliptic arteries, emphasizing the critical role of artery shape in clinical evaluations as opposed to the commonly studied circular arteries. Unlike prior work, this research offers a precise definition of stenosis by incorporating the measured length, height, and position of the narrowing. Employing the non-Newtonian Williamson fluid model, we conducted comprehensive numerical simulations to examine blood flow through four distinct stenosis formations. The novelty of this work lies in its accurate modeling of stenosis and use of advanced mesh generation, combined with commercial software and the finite volume method, to capture detailed hemodynamic behavior. Visualized results, including pressure profiles, velocity line graphs, and streamlines, further underscore the distinctive flow dynamics shaped by the elliptic geometry. Key findings of the obtained results reveal that blood velocity peaks near the stenosis and drops significantly post-stenosis, with notable variations in flow patterns, energy loss, and pressure distribution across different stenosis types. Further, higher velocity of blood flow is observed in elliptic arteries in comparison with circular ones. In the area of the high corners of stenotic segments, the pressure profile reaches high values. As a result of the narrowing of the arterial cross-section, the varied time shows that the post-stenotic segment of the artery has a higher pressure than the pre-stenotic section. The varied time suggests that an axially symmetric profile will eventually be the norm for the flow within the arterial portion. These insights have profound implications for improving clinical diagnosis and treatment strategies for conditions related to stenosed elliptic arteries.

## Introduction

Computational fluid dynamics (CFD) is a specialized branch of fluid mechanics that employs numerical methods and algorithms to solve and analyze complex fluid flow problems [1]. At its core, CFD involves the application of mathematical equations, such as the Navier-Stokes equations, which govern the conservation of mass and momentum in fluid systems. These equations describe the behavior of fluids, encompassing both liquids and gases, in response to various physical forces [2–4]. The distinctive feature of CFD lies in its ability to transform these continuous equations into discrete forms through the process of discretization, creating a computational grid that represents the fluid domain. This computational grid allows for the approximation of fluid properties at discrete points, enabling the simulation of fluid dynamics over time [5,6]. The numerical solutions generated through CFD simulations provide detailed insights into the velocity, pressure, and temperature distributions within the fluid domain [7–9]. This capability makes CFD a powerful tool in understanding and predicting the intricate behavior of fluids in different fields. The application of CFD extends across diverse industries, ranging from aerospace and automotive engineering to environmental science and biomedical research [10]. This research paper delves into the fundamental principles of CFD and explores its applications in the medical field, focusing on its role in understanding and improving medical devices and procedures.

The medical field presents a unique set of challenges that can be effectively addressed through the application of CFD. Some key areas where CFD has proven invaluable in medical research and practice include: Cardiovascular modeling: CFD is extensively used to simulate blood flow within the cardiovascular system. By understanding fluid dynamics in arteries and veins, researchers can gain insights into the development of cardiovascular diseases and optimize the design of medical devices such as stents and artificial heart valves [11]. Respiratory system analysis: CFD aids in the study of airflow patterns within the respiratory system. This is particularly useful for optimizing inhaler designs, predicting drug deposition in the lungs, and understanding the effects of diseases like asthma and chronic obstructive pulmonary disease (COPD) [12]. Drug delivery systems: CFD plays a vital role in designing and optimizing drug delivery systems. By simulating fluid dynamics in the human body, researchers can enhance the efficiency of drug distribution, leading to improved therapeutic outcomes and reduced side effects [13]. Orthopedic device design: In the realm of orthopedics, CFD is employed to analyze fluid flow and pressure distribution around joint implants. This helps in designing prosthetics and orthopedic implants that minimize wear and maximize functionality [14]. Biomedical device performance: CFD assists in evaluating the performance of various biomedical devices, such as blood pumps and dialysis machines. Understanding fluid dynamics ensures the efficient and safe operation of these devices within the human body [15]. Blood flow dynamics: CFD is used to model blood flow in arteries and veins, providing insights into hemodynamics. This is crucial for understanding the formation and progression of vascular diseases such as atherosclerosis. Additionally, CFD aids in the design of vascular implants and the assessment of blood flow patterns post-surgery [16]. Brain fluid dynamics: CFD is applied to study cerebrospinal fluid dynamics in the brain. This is essential for understanding conditions like hydrocephalus and optimizing the design of shunts used to manage fluid imbalances within the central nervous system [17]. Thermal therapy planning: In hyperthermia treatment planning, CFD assists in simulating the distribution of heat in tissues during therapeutic procedures. This is particularly relevant in cancer treatment, where controlled heating can enhance the effectiveness of radiation therapy or aid in drug delivery [18]. Intracranial pressure monitoring: CFD simulations are applied to model cerebrospinal fluid dynamics and intracranial pressure. This aids in the development of devices used to monitor and manage conditions like traumatic brain injuries and hydrocephalus [19]. Prosthetic limb comfort: CFD can be employed to analyze the interaction between prosthetic limbs and the residual limb. This helps in optimizing the design for comfort, reducing pressure points, and enhancing the overall functionality of prosthetic devices [20]. The finite volume method (FVM) is a powerful numerical technique employed in the study of blood flow through arteries aﬄicted with stenosis [21]. In the context of arterial modeling, the first step involves defining the artery’s geometry and discretizing it into smaller volumes, forming a mesh. The Navier-Stokes equations, which govern fluid dynamics, are then solved using FVM, accounting for factors such as blood viscosity, density, and specific boundary conditions that mimic the physiological environment, including inflow and outflow conditions and vessel wall properties [22]. To represent the stenosis itself, adjustments are made within the mesh, which can involve altering mesh density or employing specialized volumes to simulate the narrowing. FVM provides the means to obtain numerical solutions, with the choice of steady-state or transient analyses, offering insights into how blood flow is affected by stenosis over time [23]. The findings, which include variables such as pressure gradients, velocity profiles, and wall shear stress, add to a thorough comprehension of the hemodynamic effects of vascular stenosis. FVM simulations are a useful tool in clinical settings for determining the likelihood of thrombosis or plaque formation, supporting clinical decision-making, and optimizing treatment plans and interventions for stenosed arteries, such as stent design and interventional procedure assessment (for example, angioplasty). FVM is essential for clarifying the intricate blood flow in stenosed arteries and assisting in the creation of cutting-edge cardiovascular disease detection and treatment plans [24]. Stenosed arteries, so named because of their constriction, are a major cardiovascular health problem. They are mostly caused by atherosclerosis, which is the build-up of plaque in artery walls that causes the vessel lumen to shrink. Plaque is made up of calcium, fat, cholesterol, and other materials. In addition to smoking, hypertension causes damage to the artery walls and increases inflammation, which in turn leads to stenosis. Diabetes patients are more vulnerable because of their high blood sugar and insulin resistance. The diagnosis is made using a variety of techniques. Invasive angiography uses X-ray imaging and a contrast agent to identify stenotic regions. Non-invasive techniques like ultrasound (Doppler) [25], Computed Tomography (CT) Angiography, and Magnetic Resonance Angiography (MRA) assess blood flow and provide detailed artery images [26,27]. The first steps in treatment are lifestyle changes, such as stress reduction, exercise, good eating, and quitting smoking. Antihypertensives, antiplatelet medications, and statins are examples of medications that reduce plaque formation and manage risk factors. For severe stenosis, angioplasty with stent implantation or balloon catheterization may be necessary [28]. In more extreme cases, bypass surgery redirects blood flow using healthy vessels from other parts of the body. These treatments alleviate symptoms, reduce complications, and enhance patient outcomes in managing stenosed arteries and cardiovascular diseases. Heat transfer within blood arteries, particularly those affected by stenosis, plays a crucial role in cardiovascular health. Blood’s relatively high thermal conductivity influences heat distribution as it flows through a narrowed artery [29–31]. Metabolic processes within the body contribute to heat generation in the blood, closely linked to factors like tissue oxygen consumption. Thermal interactions extend to the arterial wall, with heat transfer occurring between the blood and the vessel’s inner lining, resulting in temperature gradients across the vessel wall [32–34]. Heat transport in stenosed arteries is commonly simulated using computational models that take into account stenosis geometry, blood flow rates, and thermal characteristics. Predicting temperature distributions and evaluating the effect on the vessel wall are made easier by this. It is clinically relevant to comprehend heat transport in stenosed arteries in order to diagnose the severity of stenosis and evaluate the risk of consequences. Thermography is one type of thermal imaging technology that may be used to see heat patterns in stenosed arteries and get insight into the dynamics of blood flow. Catheters and other localized heating techniques have therapeutic uses in managing stenosis, including facilitating medicine delivery and disrupting plaque. Current studies in this area improve therapeutic approaches for the diagnosis and treatment of cardiovascular illness and deepen our understanding of the function that heat transport plays in cardiovascular health [35]. The majority of blood flow studies that have been reported have offered precise or perturbation answers for interpreting the flow profile inside stenotic areas of the arteries. However, in situations when we must to take into account a few intricate geometrical models of arterial segments stenosis, a numerical technique to understand such issues is more trustworthy. The use of numbers to solve such issues are advantageous for taking into account complicated geometric representations of arterial stenotic segments, but in addition to is not necessary to restrict our analysis to patients with minor stenosis, etc. The research gap is addressed in this paragraph in which S. Nadeem et al. [36] research culminates the arterial blood flow analysis through distinct stenotic regions. Four different forms of stenotic regions are woven together in present study, i.e. triangular, trapezoidal, overlapping (w-shape) and composite formations. The four reviewed stenotic formations are considered in such numerical analysis by using Newtonian fluid. Elhanafy et al. [37] had numerically evaluated the hemodynamic characteristics of blood flow in arterial stenotic section with multiple degree of stenosis. Yan et al. [38] had investigated the hemodynamics rheology inside an arterial segment having cone shape of stenosis. Tripathi et al. [39] had unfolded a computational analysis on blood flow through a symmetric arterial stenotic region. Siddiqua et al. [40] had analyzed the numerical solutions of a biofluid (blood) flow inside a rectangular conduit. Carvalho et al. [41] had disclosed the numerical analysis on the blood flow within stenotic region of a coronary vessel. Although the blood is considered as a non-Newtonian fluid but in some large arteries like aorta where the shear rate is greater than one hundred per second, the blood flow shows a Newtonian nature. Therefore, blood is considered as a Newtonian nature fluid in such large arteries. But in our current issue, we have considered blood as a Williamsons fluid (non-Newtonian fluid). In present analysis, we have numerically evaluated the physics of blood flow inside multiple arterial segments. The analysis is based on blood flow through four distinct forms of stenosed arterial segments that include healthy artery, 40*%* stenosis artery, 60*%* stenosis artery and 80*%* stenosis artery stenotic regions. The free source CFD numerical software is utilized to model the blood flow through these arterial stenotic segments. No such combination of arterial stenosis segments with numerical solutions interpretation is considered in the available literature. When taking into account different forms of complicated stenosis in arteries, the numerical method is more beneficial. The Finite Element Method, which forms the foundation of commercial software for multiphysics, perfectly produces the numerical solutions of linked differential equations in part. The artery segment under examination has an elliptical cross-section, which is preserved by taking the ellipse equation into account for the boundary conditions. Consequently, the cartesian coordinate system is taken into mind when formulating the entire problem. For every instance of the examined arterial stenotic segments, the graphical solutions provide a thorough study of the flow magnitude profile , pressure profile, pressure contours, and streamlines. **Motivation of study** The primary motivation of this study is to address the limitations of previous research that predominantly focused on circular arteries, overlooking the clinical significance of arterial shape, specifically elliptic arteries, in the context of stenosis. By accurately defining stenosis through precise measurements of length, height, and position, this research aims to provide a more realistic and detailed analysis of blood flow behavior in stenosed arteries. The goal is to enhance understanding of the hemodynamic effects of stenosis in elliptic arteries, offering insights that could improve clinical diagnosis, treatment strategies, and patient outcomes in conditions related to arterial narrowing. This study also seeks to explore the non-Newtonian nature of blood flow using advanced numerical techniques and commercial software to provide a comprehensive evaluation of the changes in flow dynamics, energy loss, and pressure distribution caused by different stenosis formations.

## Mathematical formulation

**Fig 1:**
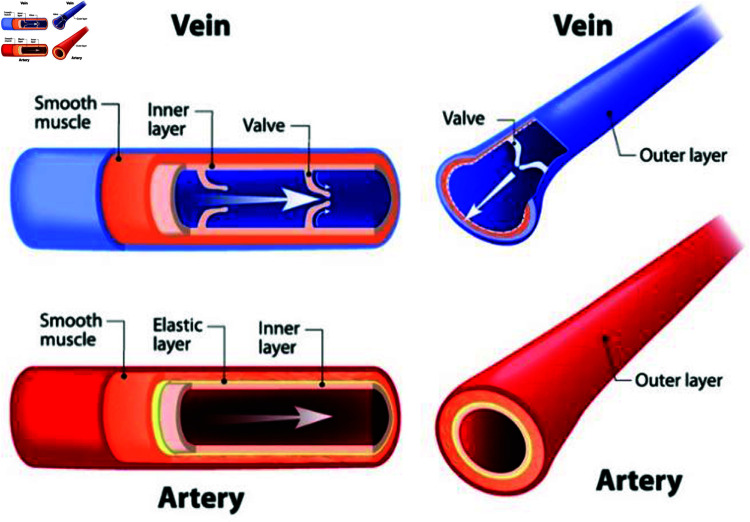
Inner and outer part of veins and arteries.

**Fig 2:**
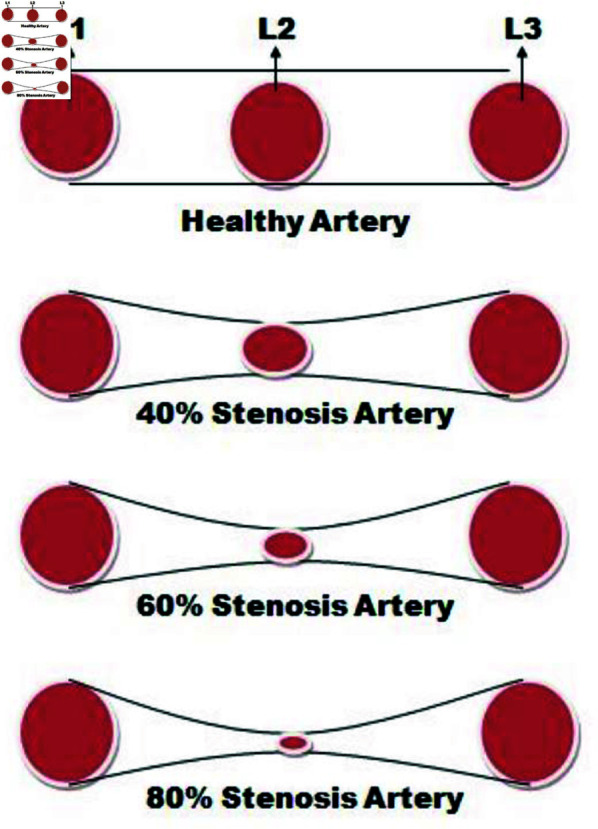
Geometry of healthy, 40*%*, 60*%* and 80*%* stenosis artery.

Let us consider laminar, unsteady, incompressible and non-Newtonian fluid flow within a 3*D* cylindrical artery as shown in Fig 1. The geometry is defined in the cartesian coordinates system *x*, *y*, and *z*-axes and comprises three circles of the same diameter representing different cross-sections: the inlet circle (*L*1), center circle (*L*2) and outlet circle (*L*3). Four distinct cases are investigated, where the fluid flows smoothly in parallel layers. The cases include a healthy artery with no stenosis, a 40*%* stenosis (40*%* reduction in the diameter of the center circle), a 60*%* stenosis, and an 80*%* stenosis as shown in Fig 2. This study employs the finite element method, a numerical technique for solving fluid flow equations is applied to analyze factors like fluid velocity, pressure, and temperature distributions. Additionally, the walls of the arteries are heated, and the specifics of the Williamson’s fluid flow model used for the modeling approach. Overall, this investigation provides insights into the impact of stenosis levels on blood flow in the arteries, which has clinical relevance for understanding cardiovascular health.

The existing blood flow problem is modeled for the non-Newtonian Williamsons fluid since it is the most extensively used model for blood flow and we can have more realistic simulation results. The governing equations for a non-Newtonian flow problem in cartesian coordinates system are given [42,43]:

**Equation of continuity**


$$\begin{array}{ccc} \frac{\partial ũ}{\partial \tilde{x}}+\frac{\partial ṽ}{\partial ỹ}+\frac{\partial \tilde{w}}{\partial \tilde{z}}=0. & & \end{array}$$ (1)

The momentum equations in cartesian coordinates system along *x*, *y* and *z*–*axis* are following:

**Momentum equation along x-axis**


$$\begin{array}{ccc} \rho_{f}\left(ũ\frac{\partial ũ}{\partial \tilde{x}}+ṽ\frac{\partial ũ}{\partial ỹ}+\tilde{w}\frac{\partial ũ}{\partial \tilde{z}}\right)= & -\frac{\partial \tilde{p}}{\partial \tilde{x}}+\nabla \cdot \left(\mu (\dot{\gamma })\nabla ũ\right), & \end{array}$$ (2)

**Momentum equation along y-axis**


$$\begin{array}{ccc} \rho_{f}\left(ũ\frac{\partial ṽ}{\partial \tilde{x}}+ṽ\frac{\partial ṽ}{\partial ỹ}+\tilde{w}\frac{\partial ṽ}{\partial \tilde{z}}\right)= & -\frac{\partial \tilde{p}}{\partial ỹ}+\nabla \cdot \left(\mu (\dot{\gamma })\nabla ṽ\right), & \end{array}$$ (3)

**Momentum equation along z-axis**


$$\begin{array}{ccc} \rho_{f}\left(ũ\frac{\partial \tilde{w}}{\partial \tilde{x}}+ṽ\frac{\partial \tilde{w}}{\partial ỹ}+\tilde{w}\frac{\partial \tilde{w}}{\partial \tilde{z}}\right)= & -\frac{\partial \tilde{p}}{\partial \tilde{z}}+\nabla \cdot \left(\mu (\dot{\gamma })\nabla \tilde{w}\right), & \end{array}$$ (4)

where, $\mu (\dot{\gamma })$ is the viscosity of the Williamson fluid and is given by:


$$\begin{array}{lll} \mu (\dot{\gamma })=\mu_{\infty }+\frac{\mu_{0}-\mu_{\infty }}{1+\lambda \dot{\gamma }}. & \; & \end{array}$$

The energy equation with viscous dissipation is following: **Energy equation**


$$\begin{array}{cccc} & \rho C_{p}(ũ\frac{\partial \tilde{T}}{\partial \tilde{x}}+ṽ\frac{\partial \tilde{T}}{\partial ỹ}+\tilde{w}\frac{\partial \tilde{T}}{\partial \tilde{z}})=k(\frac{\partial^{2}\tilde{T}}{\partial {\tilde{x}}^{2}}+\frac{\partial^{2}\tilde{T}}{\partial {ỹ}^{2}}+\frac{\partial^{2}\tilde{T}}{\partial {\tilde{z}}^{2}}) & & \\ & +\mu \alpha [2[{\left(\frac{\partial ũ}{\partial \tilde{x}}\right)}^{2}+{\left(\frac{\partial ṽ}{\partial ỹ}\right)}^{2}+{\left(\frac{\partial \tilde{w}}{\partial \tilde{z}}\right)}^{2}]+(\frac{\partial ũ}{\partial ỹ}+\frac{\partial ṽ}{\partial \tilde{x}}+\frac{\partial \tilde{w}}{\partial \tilde{z}}{)}^{2}]. & \end{array}$$ (5)

Here, *k* is called the coefficient of thermal conductivity and *α* is called the viscous dissipation parameter.

**Dimensionless variables**

The dimensionless variables for the current flow problem are [45].


$$\begin{array}{cccc} & ū=\frac{ũ}{{ũ}_{o}},\;\;\;\;\;\bar{v}=\frac{ṽ}{{ũ}_{o}},\;\;\;\;\bar{x}=\frac{\tilde{x}}{L},\;\;\;\;ȳ=\frac{ỹ}{L},\;\;\;\;\bar{p}=\frac{P}{\rho .{{ũ}_{o}}^{2}},\;\;\;\;\;\beta =\frac{k}{\rho C_{p}}, & & \\ & \text{Br}=\frac{\rho C_{p}.\mu .{ũ}_{o}^{2}}{k.({Ť}_{b}-{Ť}_{i})},\;\;\;\;\;\text{Re}=\frac{\rho .{ũ}_{o}.L}{\mu },\;\;\;\;\;\theta =\frac{Ť-{Ť}_{i}}{{Ť}_{b}-{Ť}_{i}},\;\;\;\;\;\;\text{Pr}=\frac{\rho C_{p}.\mu }{\rho .k} & & \\ & \tilde{Q}=\frac{q.L^{2}}{K_{s}.({Ť}_{b}-{Ť}_{i})},\;\;\;\;\;\bar{Q}=\frac{\rho C_{p}.q.L^{2}}{k.({Ť}_{b}-{Ť}_{i})},\;\;\;\;\;\theta_{s}=\frac{\overset{ˇ}{T_{s}}-\overset{ˇ}{T_{h_{2}}}}{{Ť}_{h_{1}}-{Ť}_{h_{2}}} & \end{array}$$ (6)

where, *θ*, Re, Br and Pr refers to the temperature, Reynoyld number, Brownian motion parameter and Prandtl number parameter respectively. The dimensionless equation of continuity, momentum equation and energy equation are following after using the dimensionless variables Eq 6 in Eqs (1), (2), (3), (4), and (5): **Equation of continuity**


$$\frac{\partial ū}{\partial \bar{x}}+\frac{\partial \bar{v}}{\partial ȳ}+\frac{\partial \bar{w}}{\partial \bar{z}}=0.$$

The momentum equations in cartesian coordinate systems along *x* , *y* and *z*–*axis* are following:

**Momentum equation along x-axis**


$$\begin{array}{llll} \frac{1}{Re}\left(ū\frac{\partial ū}{\partial \bar{x}}+\bar{v}\frac{\partial ū}{\partial ȳ}+\bar{w}\frac{\partial ū}{\partial \bar{z}}\right)= & -\frac{\partial \bar{p}}{\partial \bar{x}}+\frac{\partial }{\partial \bar{x}}\left(\frac{\mu }{\beta }\left[2{\left(\frac{\partial ū}{\partial \bar{x}}\right)}^{2}+{\left(\frac{\partial \bar{v}}{\partial ȳ}+\frac{\partial \bar{w}}{\partial \bar{z}}\right)}^{2}\right]\right),\; & & \; \end{array}$$

**Momentum equation along y-axis**


$$\begin{array}{llll} \frac{1}{Re}\left(ū\frac{\partial \bar{v}}{\partial \bar{x}}+\bar{v}\frac{\partial \bar{v}}{\partial ȳ}+\bar{w}\frac{\partial \bar{v}}{\partial \bar{z}}\right)= & -\frac{\partial \bar{p}}{\partial ȳ}+\frac{\partial }{\partial ȳ}\left(\frac{\mu }{\beta }\left[2{\left(\frac{\partial \bar{v}}{\partial ȳ}\right)}^{2}+{\left(\frac{\partial ū}{\partial \bar{x}}+\frac{\partial \bar{w}}{\partial \bar{z}}\right)}^{2}\right]\right),\; & & \; \end{array}$$

**Momentum equation along z-axis**


$$\begin{array}{llll} \frac{1}{Re}\left(ū\frac{\partial \bar{w}}{\partial \bar{x}}+\bar{v}\frac{\partial \bar{w}}{\partial ȳ}+\bar{w}\frac{\partial \bar{w}}{\partial \bar{z}}\right)= & -\frac{\partial \bar{p}}{\partial \bar{z}}+\frac{\partial }{\partial \bar{z}}\left(\frac{\mu }{\beta }\left[2{\left(\frac{\partial \bar{w}}{\partial \bar{z}}\right)}^{2}+{\left(\frac{\partial ū}{\partial \bar{x}}+\frac{\partial \bar{v}}{\partial ȳ}\right)}^{2}\right]\right),\; & & \; \end{array}$$

**Energy equation**


$$\begin{array}{llll} (ū\frac{\partial \bar{\theta }}{\partial \bar{x}}+\bar{v}\frac{\partial \bar{\theta }}{\partial ȳ}+\bar{w}\frac{\partial \bar{\theta }}{\partial ȳ})= & \frac{1}{\text{Re}.\text{Pr}}(\frac{\partial^{2}\bar{\theta }}{\partial {\bar{x}}^{2}}+\frac{\partial^{2}\bar{\theta }}{\partial {ȳ}^{2}}+\frac{\partial^{2}\bar{\theta }}{\partial {\bar{z}}^{2}})\; & & \;\\ & +\frac{\text{Br}}{\text{Re}.\text{Pr}}[2[{\left(\frac{\partial ū}{\partial \bar{x}}\right)}^{2}+{\left(\frac{\partial \bar{v}}{\partial ȳ}\right)}^{2}+{\left(\frac{\partial \bar{w}}{\partial \bar{z}}\right)}^{2}]+(\frac{\partial ū}{\partial ȳ}+\frac{\partial \bar{v}}{\partial \bar{x}}+\frac{\partial \bar{w}}{\partial \bar{z}}{)}^{2}].\; & & \; \end{array}$$

The stress tensor in Williamson’s fluid, a non-Newtonian fluid model, captures the complex relationship between stress and strain rate, specifically accounting for shear-thinning behavior. Unlike Newtonian fluids, where stress is linearly proportional to the strain rate, Williamson’s fluid exhibits a nonlinear relationship that diminishes with increasing shear rates. The fluid’s viscosity decreases as the applied stress increases, leading to a more gradual response in high-shear regions, such as near stenosis in blood flow. Mathematically, the stress tensor incorporates this nonlinearity by modifying the viscosity term with parameters that characterize the fluid’s yield stress and relaxation time, reflecting the fluid’s ability to transition between Newtonian-like behavior at low shear and reduced viscosity at high shear. This model is particularly effective in simulating biological fluids like blood, where such shear-thinning effects are crucial for accurately predicting flow behavior and stress distribution under varying physiological conditions.

The considered extra stresses tensor is addressed in Eq (7) for Williamsons fluid model [44].


$$\begin{array}{ccc} \delta_{ij}=\eta_{o}(1+\Gamma |\gamma_{ij}|)\gamma_{ij}, & & \end{array}$$ (7)

here,


$$\begin{array}{llll} & \gamma_{ij}=\frac{\partial \upsilon_{i}}{\partial x_{j}}+\frac{\partial \upsilon_{j}}{\partial x_{i}},\; & & \;\\ & |\gamma_{ij}|=\sqrt{(\frac{1}{2}\underset{i}{\sum }\underset{j}{\sum }\gamma_{ij})}.\; & & \; \end{array}$$

During the computational fluid dynamics approach by using ANSYS software, the main boundary conditions applied for the healthy, 40%, 60%, and 80% stenosed arteries for velocity and temperature profiles typically include:

- **Inlet boundary condition:** 1. Velocity Profile: A fully developed laminar flow or parabolic velocity profile is applied at the inlet. The velocity is specified based on physiological blood flow conditions. 2. Temperature Profile: A constant temperature or a heat flux condition may be applied at the inlet to simulate thermal behavior.
- **Outlet boundary condition:** 1. Pressure Outlet: A constant pressure or zero gauge pressure is often applied at the outlet to simulate the blood leaving the artery without resistance.
- **Wall boundary condition:** 1. No-Slip Condition: The artery walls are modeled with a no-slip boundary condition, meaning the velocity of the fluid at the wall is zero. 2.Thermal Condition: For temperature profiles, adiabatic (no heat flux) or constant wall temperature conditions can be applied at the artery walls, depending on the simulation’s focus.
- **Symmetry boundary condition:** If the model uses symmetry to reduce computation, a symmetry boundary condition is applied to the appropriate surfaces to ensure accurate flow and thermal results without recalculating redundant sections.

These boundary conditions are selected to closely replicate physiological conditions in stenosed arteries and analyze the velocity and temperature profiles under different stenosis levels. **Boundary conditions** The above associated boundary conditions are used in simulations and modeling for the results of velocity, heat and pressure and are given in Table 1 as:

**Table 1:** Boundary conditions for 3-D problem.

Conditions	Flow Equations	Heat Equations
Wall	*u* = *v* = *w* = 0	*θ* = *given* , *n* × *q* = *given* , *n* . *q* = *given*
Specified inlet (outlet)	*u* = *given* , *v* = *given* , *w* = *given*	*θ* = *given* , *n* × *q* = *given* , *n* . *q* = *given*
Symmetry	*n* . *u* = 0 , *n* × *w* = 0	*n* . *q* = 0

## Meshing

Meshing is the process of approximating the geometry and enabling the numerical solution of physical problems in domains such as computational fluid dynamics and finite volume analysis. The smaller, discrete volumes that are used in meshing are triangles or quadrilaterals in 2*D* or tetrahedra and hexahedra in 3*D*. In order to apply boundary conditions, adapt to particular regions of interest, and perform post-processing to extract results and visualize the simulated systems all of which have an impact on the accuracy and computational efficiency of the analysis meshing is essential for precise and effective simulations.

**Fig 3:**
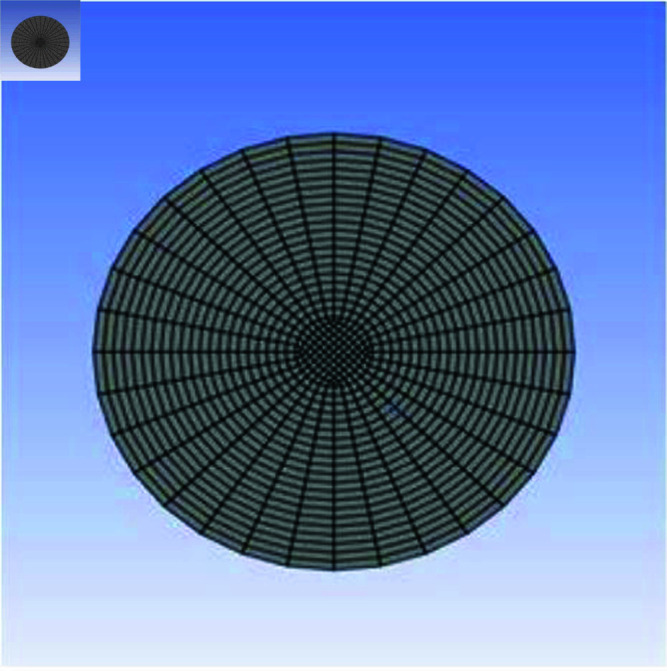
Front meshing representation of artery.

In Fig 3, the intima, media, and adventitia are the three separate layers that make up a blood artery’s unique cylindrical form when viewed from the front. From this vantage point, the arteries’ pulsating behavior a result of their elastic properties and their complex branching network, which acts as the body’s lifeblood highway, are exquisitely displayed. It is noteworthy because it emphasizes the crucial idea that when arteries narrow and split, their cross-sectional area must also decrease. This is essential for preserving accurate blood pressure management. Not to be overlooked are the endothelial cells that line the inside of arteries, which are in charge of controlling blood flow patterns and preventing clot formation. This viewpoint is crucial from a clinical perspective when it comes to cardiovascular health because it provides a special perspective for evaluating arterial health, identifying blockages or irregularities, and precisely navigating interventional procedures in the field of cardiovascular medicine.

**Fig 4:**
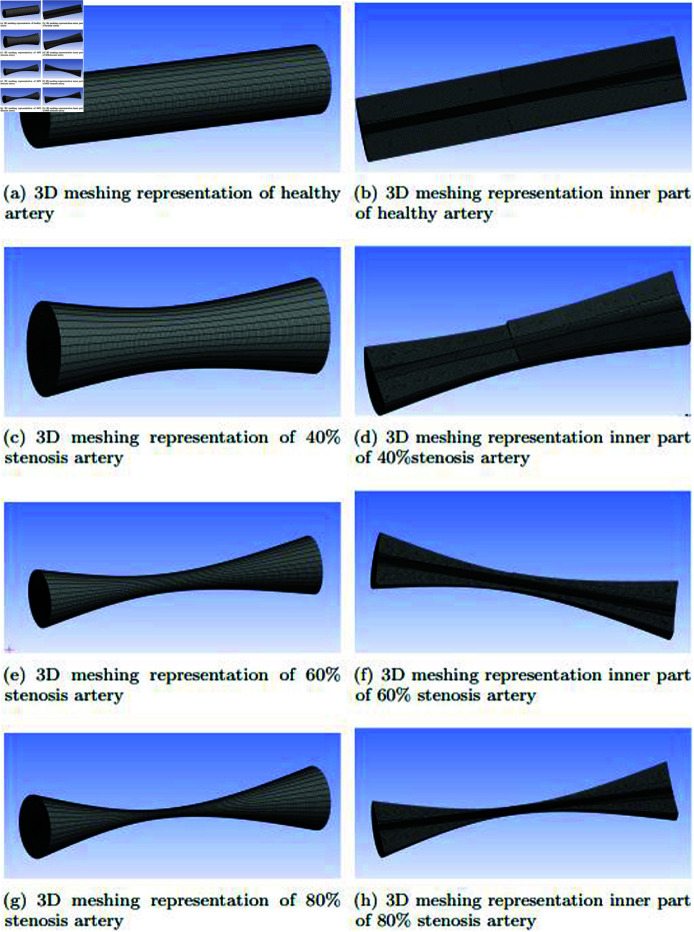
3D meshing representation of healthy, 40*%*, 60*%* and 80*%* stenosis artery.

In Fig 4(a) and 4(b), a rigorous mesh independence test is used to carefully evaluate the mesh representation. The mesh is made to provide a very high degree of accuracy while looking at a healthy artery. With 197 , 547 nodes and 192 , 544 components overall, the mesh exhibits impressive detail in this setting, highlighting the intricate and complicated structural intricacy of the artery. This degree of mesh representation precision is essential for accurately modeling and simulating the behavior of arteries, especially in situations where precision and attention to detail are critical, like in computational fluid dynamics simulations of blood flow in vascular systems or biomedical research. In Fig 4(c) and 4(d), the mesh depiction keeps stressing accuracy while considering an artery with a *k* + 1 stenosis. It catches the details of the somewhat constricted artery anatomy with great care, exposing 149 , 824 components overall and 154,763 nodes. The accuracy with which stenosis is simulated and analyzed a condition in which the artery narrows as a result of plaque accumulation or other factors requires this degree of precision in the mesh. In the domain of medical modeling and computational simulations, such mesh representation accuracy is especially useful for evaluating blood flow variations, pressure differentials, and possible therapeutic actions targeted at treating stenotic problems in arterial health. In Fig 4(e) and 4(f), within a 60*%* stenosis artery, the inner part presents a notably altered and constricted geometry. The mesh representation, composed of 133 , 118 nodes and a total of 108 , 224 volumes, offers an intricate view of this narrowed arterial segment. Such meticulous detailing is indispensable for accurately simulating the complex blood flow patterns, pressure gradients, and the hemodynamic impact of a substantial stenosis in biomedical research and clinical contexts. It aids in a deeper understanding of the vascular condition and guides potential interventions to address the health implications of this level of stenotic severity. In the Fig 4(g) and 4(h), the inner portion of the arterial shows a remarkably restricted and drastically changed geometry in an artery with 80*%* stenosis. With 97 , 583 nodes and 92 , 640 volumes overall, the mesh representation employed to simulate this crucial region offers an incredibly precise picture of the extreme artery constriction. For the purpose of precisely replicating the complicated hemodynamic changes, blood flow interruptions, and pressure differentials associated with such an advanced stenotic disease, this degree of precision is crucial. It is essential for research and therapeutic applications, allowing a thorough comprehension of the consequences for arterial health and directing possible therapies for cases of severe stenosis. As stenosis, or the narrowing of these blood vessels, worsens, it is easy to see that the cross-sectional area of the arteries decreases. The effect of the stenosis on the artery lumen directly leads to this decrease in cross-sectional area. The amount of nodes and components used in the mesh representation of these arteries reduces as the degree of stenosis increases. The stenotic condition’s severely constrained and reduced geometry is responsible for this reduction in mesh complexity. Essentially, it represents the development of vascular disease and is an essential component of effectively simulating, in both research and clinical settings, the hemodynamic alterations and flow disturbances linked to more severe stenosis.

### Mesh independence test anaylsis

**Fig 5:**
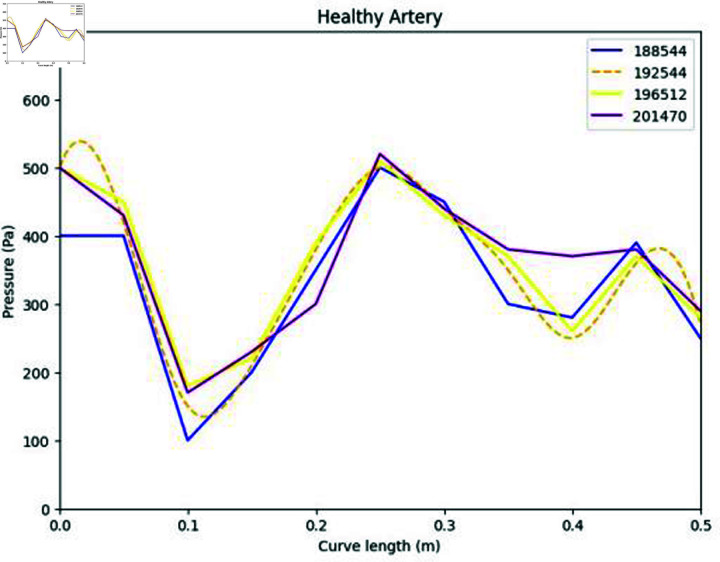
Representation of mesh grid and grid independence test analysis.

The accuracy and reliability of numerical simulations are heavily influenced by the mesh structure and the implementation of a grid independence test and graphically as shown in Fig 5. In this study, the computational domain, representing a healthy artery, was discretized using a multizone meshing technique. This method ensures better convergence and stability in solving the governing equations. A grid independence test was conducted to validate that the results are not sensitive to the mesh size. Simulations were performed with various mesh elements (188,544, 192,544, 196,512, and 201,470) while maintaining a constant Reynolds number. The average pressure values were plotted, revealing that the solution converges at 192,544 elements as shown in Fig 4. This confirms that beyond this point, further mesh refinement does not significantly affect the results, ensuring the solution’s robustness and independence from mesh size.

## Results and discussion

### Case: 01 (For healthy artery)

**Fig 6:**
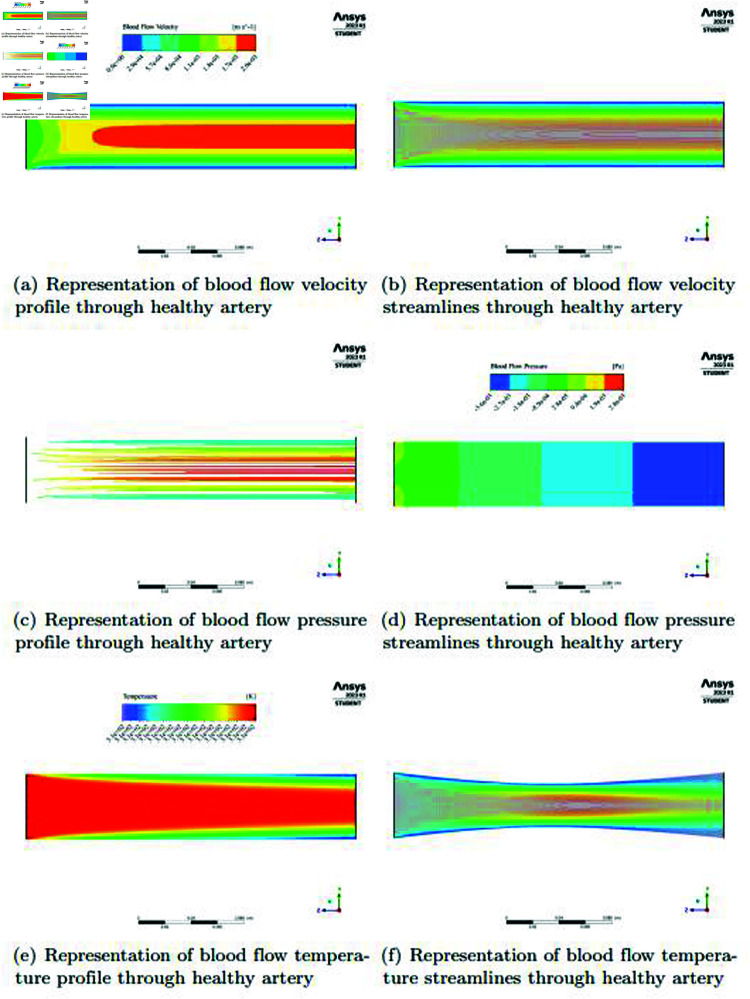
Representation of blood flow velocity, pressure, temperature and streamlines profiles through healthy artery.

In the Fig 6(a) and 6(b), in a healthy artery, blood flow has a characteristic velocity profile, with the greatest velocities concentrated in the lumen’s center. The formation of boundary layers along the artery walls is the cause of this occurrence. Studies have indicated that the frictional contact between the stationary artery walls and the flowing blood causes these boundary layers to form. Blood velocity is therefore highest in the very core of the artery, where the influence of these border layers is least. However, the growing effect of these boundary layers causes the velocity to steadily decrease as one advances from the center towards the artery walls. Knowledge blood flow dynamics and how it affects vascular health requires a knowledge of this distinctive flow profile, which is a critical component of hemodynamics and influences the distribution of shear forces and pressure gradients throughout the vascular system. The visualization of streamlines is used in studies on healthy arteries to give a more exact and accurate depiction of blood flow dynamics. These streamlines provide a useful illustration of the artery’s velocity characteristics. The streamlines in a healthy artery usually show a central core where blood flows at its fastest. The flow patterns in this center core are streamlined and have little disruption. The streamlines that surround this core show a progressive slowing down as they get closer to the vessel walls. The reason for this decrease in speed is the development of boundary layers close to the artery walls, which is a result of friction between the blood and the inside surface of the channel. By helping researchers observe and study the distribution of velocities and shear forces two critical volumes in preserving arterial health and function, the use of streamlines improves our knowledge of the complex flow dynamics in healthy arteries.

In the Fig 6(c) and 6(d), the profiles of pressure and velocity are inversely correlated. Studies on the hemodynamics of blood vessels have extensively reported this phenomena. near particular, the artery’s velocity profile reveals that the blood flows most smoothly near the center of the lumen, where the highest velocities are found. There is less pressure in this core area. In contrast, the drag impact of boundary layers causes the surrounding regions, especially those close to the artery walls, to show lower velocities. As a result, there is more pressure in these areas. Pressure progressively drops as one approaches the center of the artery from its perimeter. A vital component of healthy arterial blood flow, this pressure gradient ensures effective circulation and oxygen delivery to the body’s tissues. In the Fig 6(e) and 6(f), there is a noticeable temperature profile in a healthy artery. The blood temperature is comparatively greater at the artery’s beginning, nearer the heart. This is because the body’s metabolic processes produce heat, which heats the blood as it is pushed out of the heart. The temperature equalizes and stabilizes as the blood travels through the artery and into the middle of the vessel. The temperature distribution in this core section of the artery is often more consistent. This thermal equilibrium, which reflects the complex balance of physiological activities inside the circulatory system, is necessary to maintain ideal conditions for the transport of oxygen and nutrients to the body’s tissues and organs. In order to precisely and accurately visualize the movement of thermal energy inside the circulatory system, researchers frequently use streamlines to illustrate the system. Heat transfer streamlines offer a thorough understanding of the temperature distribution along the artery in combination to velocity streamlines, which clarify blood flow patterns. These heat transfer streamlines show how thermal energy travels down the artery, moving from areas of greater temperature typically around the heart to areas of lower temperature. With the use of this visualization tool, we may better comprehend heat dispersion and the thermal dynamics that are necessary to keep the vascular system’s temperature environment steady and consistent, which guarantees effective oxygen delivery and overall physiological balance.

### Case: 02 (For  < 0 . 1 stenosis artery)

**Fig 7:**
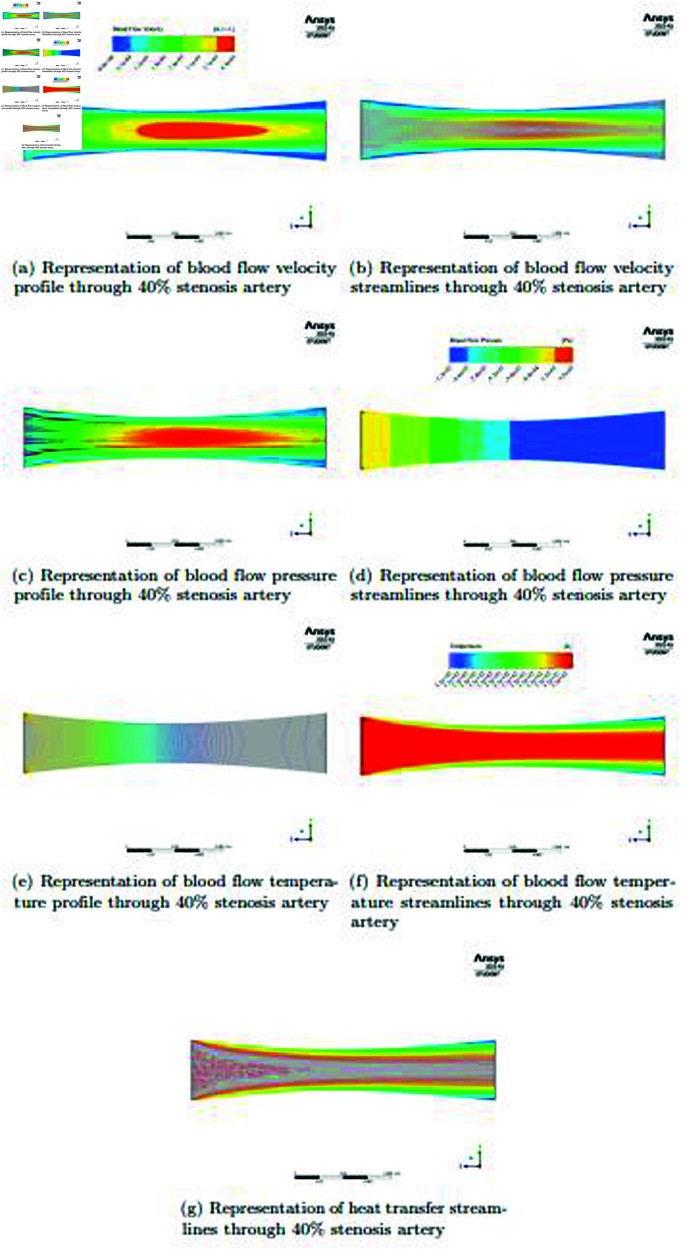
Representation of blood flow velocity, pressure, temperature, heat transfer and streamlines profiles through 40*%* stenosis artery.

In the Fig 7(a) and 7(b), when considering an artery that has  > 0 . 1 stenosis in its middle region, there is a discernible change in the velocity profile when compared to a healthy arterial. The flow dynamics are markedly altered in the presence of stenosis, which is characterized by the narrowing of the artery lumen. The artery’s cross-sectional area decreases with stenosis, significantly altering the velocity distribution. More specifically, very little blood flows forward and there is a significant reduction in blood flow near the artery’s core. The streamlines, which offer a more thorough view of the modified blood flow patterns brought on by the stenotic condition, make this shift in velocity clearly evident. In the Fig 7(c) and 7(d), an examination of blood flow pressure within an artery with 40*%* stenosis reveals a unique pattern. Interestingly, the pressure distribution shows a larger value around the stenotic area and at the artery’s initial corner positions along its walls. This finding can be explained by the fact that pressure and velocity have an inverse relationship, with slower flow brought on by stenosis often translating into higher pressure. Pressure progressively drops when the stenosed artery’s central region experiences an acceleration of flow. The streamlines effectively illustrate this dynamic interplay between pressure and velocity, providing a thorough picture of the complex effects of pressure on blood flow in the setting of stenosis. These discoveries are critical to comprehending the hemodynamic effects of arterial stenosis, illuminating possible clinical repercussions and emphasizing the significance of vascular health evaluations. In the Fig 7(e), 7(f) and 7(g), an important source of information on the mechanics of heat transport in the circulatory system is the blood flow temperature profile. Heat transmission becomes increasingly noticeable when blood starts to pass through the artery, particularly in areas close to the arterial entrance site. Nevertheless, as the blood flow reaches the stenotic section, a noticeable change in the heat transfer pattern is seen. Heat transmission in the stenosis is significantly less than in the early phases of flow. The stenotic region’s altered flow dynamics and decreased velocity are responsible for this reduction in heat transmission. Surprisingly, while the blood flows through the stenosis, the heat transfer is very steady and constant, demonstrating the preservation of thermal equilibrium. The use of streamlines contributes to the improved accuracy and lucidity of these results by offering a comprehensive and precise depiction of the heat transfer mechanism in the artery with 40*%* stenosis. This all-encompassing method has important implications for vascular health research as well as therapeutic applications. It helps to comprehend the intricate heat dynamics within stenotic arteries.

### Case: 03 (For 60*%* stenosis artery)

**Fig 8:**
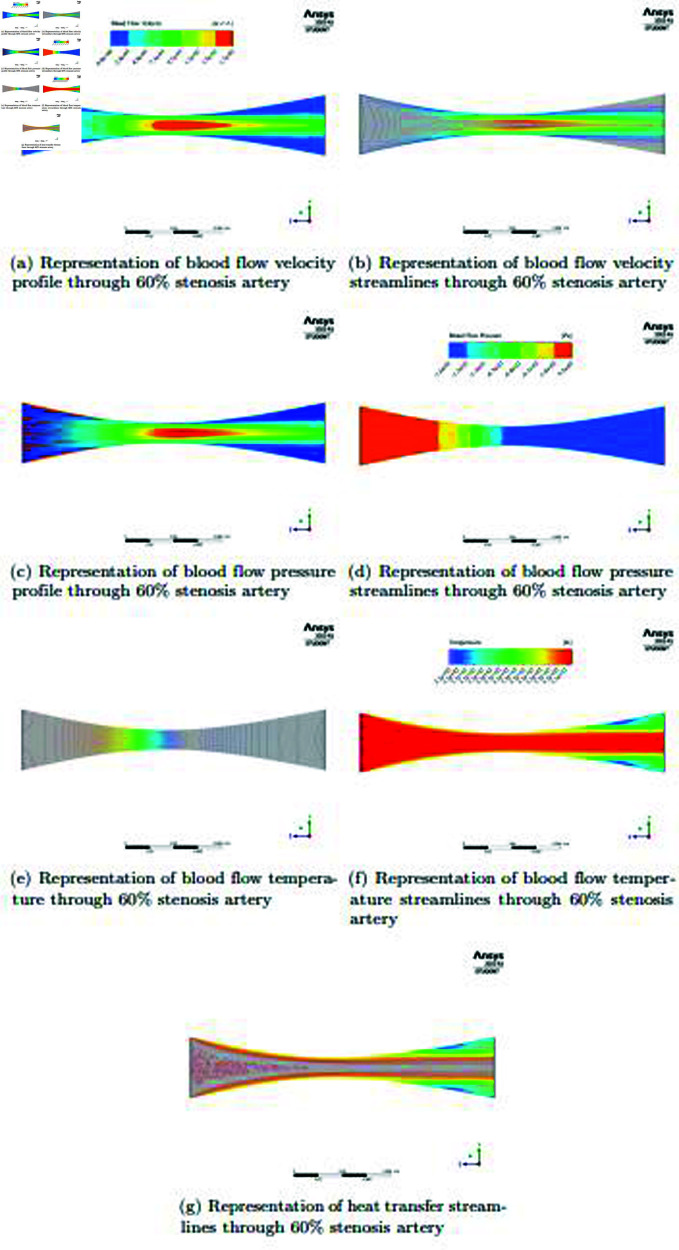
Representation of blood flow velocity, pressure, temperature, heat transfer and streamlines through 60*%* stenosis artery.

In the Fig 8(a) and 8(b), an analysis of the blood flow velocity profile in the presence of a 60*%* stenosis in the artery demonstrates a unique modification in contrast to the 40*%* stenosis artery. A smaller cross-sectional area is the outcome of a more severe stenosis, which is defined by a larger constriction of the artery lumen. As a result, just a little amount of blood is forced forward and the velocity inside the stenotic area significantly decreases. Using streamlines to show the effects of this severe stenosis on blood flow dynamics allows for a more accurate and exact depiction of these complex flow patterns. This all-inclusive method helps clarify the hemodynamic effects of progressive artery stenosis and its possible clinical ramifications for evaluations and treatments related to vascular health. In the Fig 8(c) and 8(d), a detailed analysis of blood flow pressure profiles in the setting of an artery with 60*%* stenosis identifies a noteworthy pattern. Blood pressure is highest in the artery’s beginning portion, where stenosis has caused the velocity to be noticeably reduced. On the other hand, the blood flow velocity progressively rises as it moves closer to the stenotic zone. According to the inverse relationship between velocity and pressure in fluid dynamics, this increase in velocity corresponds to a drop in pressure. A major aspect of stenosis is the dynamic interplay between pressure and velocity, which is seen in the pressure distribution along the artery. These discoveries have significance for comprehending and controlling vascular health as well as providing insightful information about the intricate hemodynamic effects of progressive artery stenosis. The addition of pressure streamlines to this image improves our comprehension and visualization of the dynamics of blood flow pressure in an artery with 60*%* stenosis. These streamlines provide significant geometrical insights on the behavior of pressure inside the artery lumen, which are essential for a more precise and thorough portrayal. We learn more about how pressure varies across the stenotic zone and the impact of velocity fluctuations by analyzing the complex patterns these streamlines create. In the Fig 8(e), 8(f) and 8(g),The heat transmission rate in the artery is shown, and some tendencies are evident. The rate of heat transmission is noticeably higher near the beginning of the artery, where blood flow begins. Nevertheless, the heat transfer rate progressively falls as the blood moves ahead and experiences the consequences of a  ≥  stenosis and the ensuing decrease in the cross-sectional area. A more uniform and smoother pattern of heat transmission inside the stenotic area indicates the change. This graphic illustrates how stenosis affects blood flow’s thermal dynamics and emphasizes how important it is to keep the vascular system’s thermal balance. These discoveries advance our knowledge of heat dispersion and have practical and scientific ramifications for vascular health-related issues. Heat transfer streamlines are included in this image as a useful tool to improve the visibility and accuracy of the heat transfer dynamics in the artery with a  <  stenosis. The distribution and transport of heat energy inside the stenotic artery are depicted in great detail by these streamlines. Researchers may learn more about the intricate thermal patterns, how stenosis affects heat distribution, and how the vascular system maintains thermal equilibrium by looking at these heat transfer streamlines.

### Case: 04 (For 80*%* stenosis artery)

**Fig 9:**
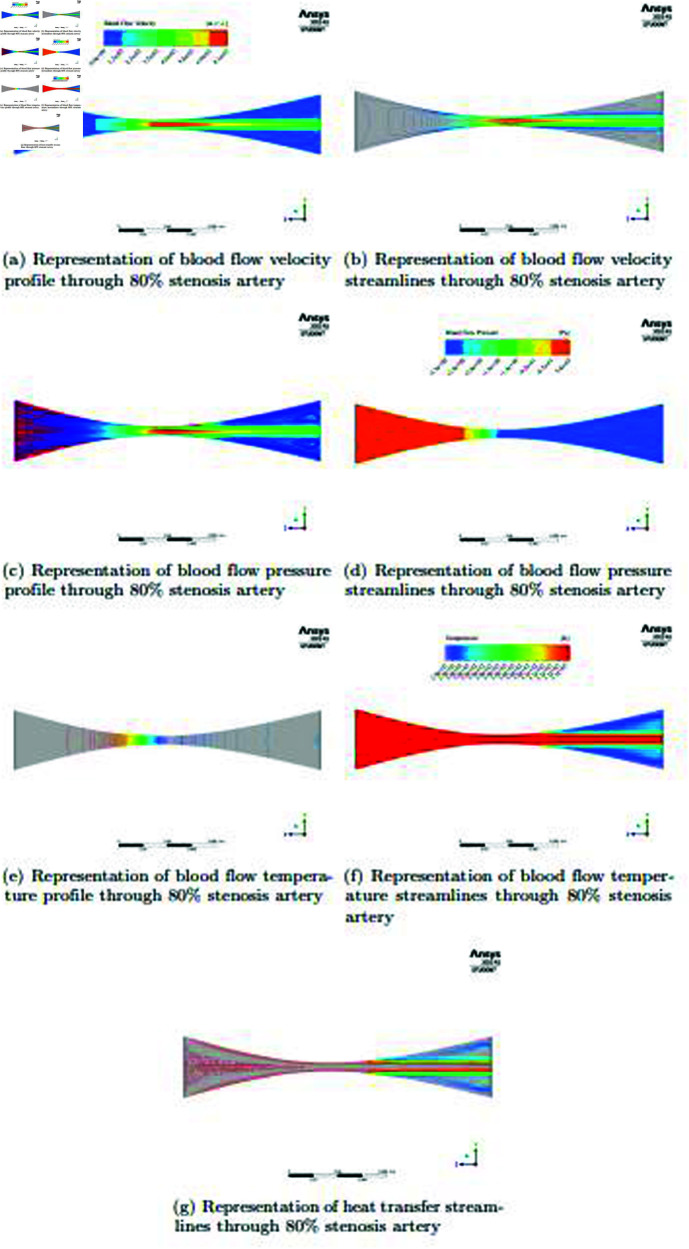
Representation of blood flow velocity, pressure, temperature, heat transfer and streamlines through 80*%* stenosis artery.

In the Fig 9(a) and 9(b), a significant reduction in cross-sectional area occurs when an artery has an 80*%* stenosis; this effect is significantly more noticeable than in prior arterial states. Interestingly, this extreme stenosis raises blood flow velocity in the artery considerably. But because of the widening, very little blood flows forward; instead, most of the blood swirls in a circular pattern inside the stenotic area. Reduced velocity is the result of this impact at the pre-post locations and along the artery wall borders. The flow dynamics are significantly affected by the presence of such severe stenosis, and these data highlight the difficulties in preserving a balanced hemodynamic profile in arteries with advanced stenosis. Comprehending these dynamics is vital for the evaluation and treatment of vascular well-being in medical settings. Streamlines are used as a useful tool to visually represent the blood flow dynamics in the artery, including the stenotic area. The various flow patterns that occur as blood passes through the artery and is affected by the 80*%* stenosis are depicted in depth by these streamlines. Through the analysis of these streamlines, scholars may get a more profound comprehension of the intricate flow dynamics, encompassing the undulating pattern seen in the stenotic section. In the Fig 9(c) and 9(d), the distribution of blood pressure is significantly affected by the artery’s 80*%* stenosis. Blood pressure is greater at the beginning of the artery where the stenosis has caused the velocity to be significantly reduced. This is a direct outcome of the inverse connection between pressure and velocity, which states that higher pressure is usually correlated with slower blood flow. On the other hand, pressure drops in the stenosis area when blood flow velocity is noticeably higher than in other areas. One important aspect of the pressure distribution along the artery is the dynamic interaction between pressure and velocity. These findings highlight the substantial hemodynamic alterations brought about by severe artery stenosis, highlighting the importance of this condition in relation to evaluations of vascular health and possible therapeutic measures. In the Fig 9(e), 9(f) and 9(g),The heat transfer rate diagram in an artery with an 80*%* stenosis shows different behavior. The heat transfer rate rises noticeably when the blood flows through the area of stenosis. This is explained by the stenotic segment’s narrowed flow channel and increased blood velocity, which promote more effective heat transfer. After the stenosis area, the bloodstream’s heat transmission rate steadies and continues without interruption. The influence of severe arterial stenosis on thermal dynamics and the preservation of thermal equilibrium in the vascular system is shown by this picture. In the context of vascular health, these discoveries are crucial for comprehending heat dispersion and have ramifications for clinical and scientific applications. Heat transfer flow streamlines are used to achieve a better degree of accuracy and precision while analyzing the dynamics of heat transfer inside the artery, particularly when there is an 80*%* stenosis. The distribution and transmission of thermal energy as blood passes through the artery, including the stenotic portion, is depicted in detail by these heat transfer streamlines. Researchers can gain a deeper understanding of the intricate thermal patterns.

### Graphically discussion of line graph for velocity magnitude

**Fig 10:**
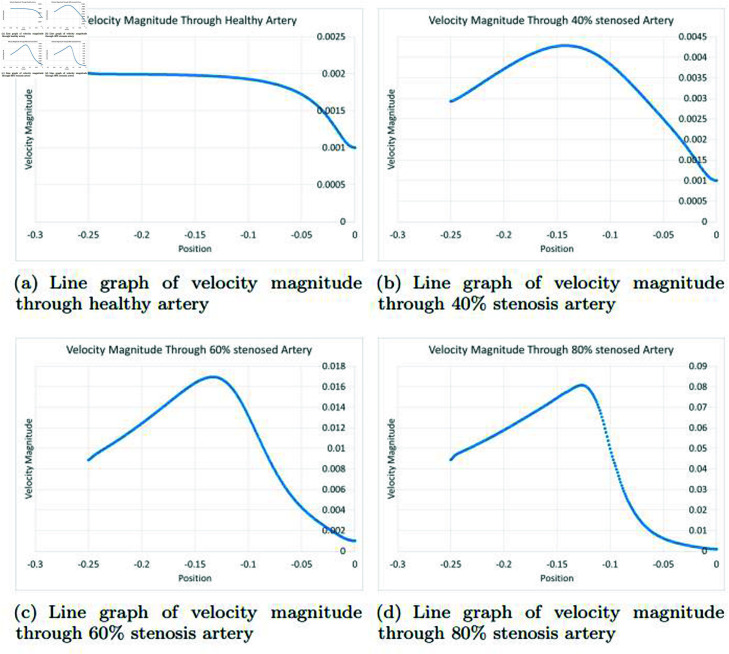
Representation of line graphs for velocity magnitude through healthy, 40*%*, 60*%* and 80*%* stenosis artery.

In the Fig 10 (a), in a velocity line graph of a healthy artery, a consistent initial flow is observed, starting at a velocity of 0.002. This signifies that the blood moves smoothly through the artery during the initial segment, possibly due to a wide and straight section of the vessel, facilitating streamlined flow. However, after a certain distance, a transition point is reached where the velocity decreases to 0.001. Several factors can contribute to this change, including alterations in vessel geometry, variations in blood viscosity, the development of friction due to plaque accumulation, and the potential for turbulence in narrower or bending sections of the artery. Despite the decrease in velocity, the graph stabilizes at 0.001, indicating that the blood flow remains relatively consistent beyond the transition point, suggesting effective circulation. In the Fig 10(b), in a velocity line graph representing a 40*%* artery blockage, a distinctive pattern emerges as blood flows through the partially occluded artery. Initially, the graph displays an upward trend in velocity, with an initial velocity of 0.002. This initial increase in velocity might be attributed to the artery’s ability to compensate for the partial blockage by dilating or by having a relatively unobstructed entry point. However, as the blood travels further along the artery, it encounters the constricted region affected by the 40*%* blockage, resulting in a significant decrease in velocity to 0.001. The blockage hinders the smooth flow of blood, causing resistance and turbulence. This decline in velocity after the initial increase signifies the artery’s reduced ability to efficiently deliver blood. The change in velocity from upward to downward is a characteristic feature of an artery with partial blockage, and it can have clinical implications. It indicates that while blood flow might initially adapt to the blockage, it eventually experiences a decrease in velocity, which can lead to inadequate blood supply to downstream tissues, potentially causing health issues. In such cases, medical intervention may be necessary to address the arterial blockage and restore normal blood flow. In the Fig 10(c), in a velocity line graph representing a 60*%* artery blockage, a distinct pattern emerges as blood flows through the significantly narrowed artery. Initially, the graph shows a more pronounced upward trend in velocity compared to a 40*%* blockage, starting with a velocity of 0.002. This initial increase in velocity could be due to the artery’s attempt to compensate for the greater blockage by dilating or by having a relatively unobstructed entry point. However, as the blood progresses through the artery, it encounters the severely constricted region affected by the 60*%* blockage, leading to a substantial decrease in velocity down to 0.001. The presence of a 60*%* blockage creates substantial resistance to blood flow, causing significant turbulence and reduced velocity. The change from initially upward to downward velocity is a key indicator of a severely blocked artery. It signifies a reduced ability of the artery to efficiently deliver blood, which can have critical clinical implications. This decrease in velocity can result in inadequate blood supply to downstream tissues, potentially leading to serious health consequences. In such cases, immediate medical intervention is often necessary to address the arterial blockage and restore proper blood flow to prevent further complications. In the Fig 10(d), in a velocity line graph of a 80*%* artery blockage, the blood flow initially experiences a more pronounced increase in velocity, starting at 0.002, compared to a 60*%* blockage. However, as the blood progresses through the artery, it encounters the severely constricted region affected by the 60*%* blockage, leading to a significant decrease in velocity down to 0.001. This shift from initially upward to downward velocity indicates a severely blocked artery, causing turbulence and reduced blood supply, which can have serious health implications. Immediate medical intervention is often necessary to address the arterial blockage and restore proper blood flow.

### Graphically discussion of line graph for radial velocity

**Fig 11:**
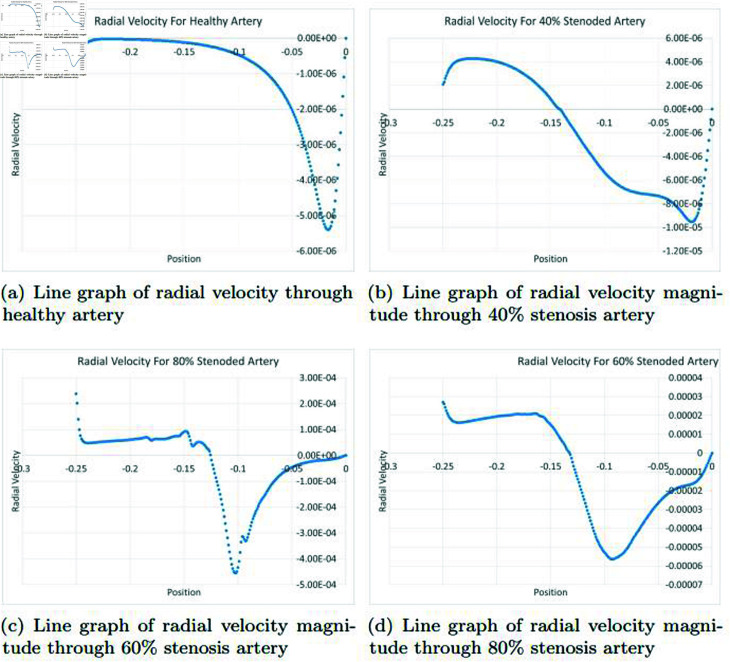
Representation of line graphs for radial velocity through healthy, 40*%*, 60*%* and 80*%* stenosis artery.

In the Fig 11(a), the representation of radial velocity in a healthy artery provides valuable insights into blood flow dynamics. Radial velocity is the component of blood velocity that moves outward from the center of the artery, perpendicular to its axis. When observing the velocity curve in a healthy artery, a distinctive pattern emerges. Initially, the curve shows a smooth and consistent upward movement, signifying organized blood flow towards the artery wall. This smooth flow is characteristic of laminar flow within the undisturbed section of the artery, where blood layers move in an orderly fashion. As the blood travels along the artery, after some distance, the radial velocity curve may exhibit a downward trend. Several factors can contribute to this decline. Changes in the artery’s geometry, such as narrowing or branching, can disrupt the smooth flow and result in decreased radial velocity as blood encounters resistance and turbulence. In the Fig 11(b), the representation of radial velocity in a 40*%* occluded artery offers insights into how blood flow is affected by a significant blockage within the artery. When examining the velocity curve, a distinct pattern emerges. Unlike a healthy artery with a smooth and consistent velocity pattern, in a 40*%* occluded artery, the radial velocity curve typically displays a downward trend followed by an upward movement. The initial downward movement in the radial velocity curve can be attributed to the presence of the 40*%* arterial blockage. This blockage creates resistance and turbulence within the artery, causing the blood to slow down and reducing the radial velocity. The narrowing of the artery results in an obstacle for the blood flow, hindering its ability to move efficiently towards the artery wall. Following this initial decline, the upward movement in the radial velocity curve may occur as the blood adapts to the altered conditions. This could be due to various factors, such as the artery’s dilation in response to the blockage or changes in blood viscosity. While the velocity may increase somewhat, it may not reach the same levels as in a healthy artery, as the blockage still presents a significant impediment to the flow. In the Fig 11(c), the representation of radial velocity in a 60*%* occluded artery provides insights into the complex effects of a severe blockage on blood flow patterns. In a 60*%* occluded artery, the radial velocity curve often displays a more intricate behavior, including a downward trend followed by an upward movement, as well as a sinusoidal or random pattern. The initial downward movement in the radial velocity curve can be primarily attributed to the severe blockage’s presence. This extensive blockage significantly disrupts the smooth and laminar flow of blood, leading to resistance and turbulence within the artery. The narrowing of the artery creates a substantial obstacle to the blood flow, causing it to decelerate and reducing the radial velocity. This downward trend is characteristic of the reduced efficiency in delivering blood to the artery wall. The subsequent upward movement, although it may occur, might not fully restore the velocity to healthy levels. Factors such as vessel dilation, changes in blood viscosity, or fluctuations in the blockage itself can contribute to this upward shift. However, the blockage’s severity means that even after this adjustment, the blood flow may still be suboptimal. The sinusoidal or random patterns seen in the radial velocity curve can be due to the turbulence and chaotic flow behavior caused by the 60*%* blockage. In the Fig 11(d), in a 80*%* occluded artery, the radial velocity curve exhibits a complex pattern. Initially, the curve moves downward due to the severe blockage’s resistance and turbulence. Subsequent upward and sinusoidal/random movements reflect attempts to adapt to the blockage’s dynamic and chaotic effects on blood flow, underscoring the challenges of severe arterial occlusion and its impact on blood supply to downstream tissues.

### Graphically discussion of line graph for static pressure

**Fig 12:**
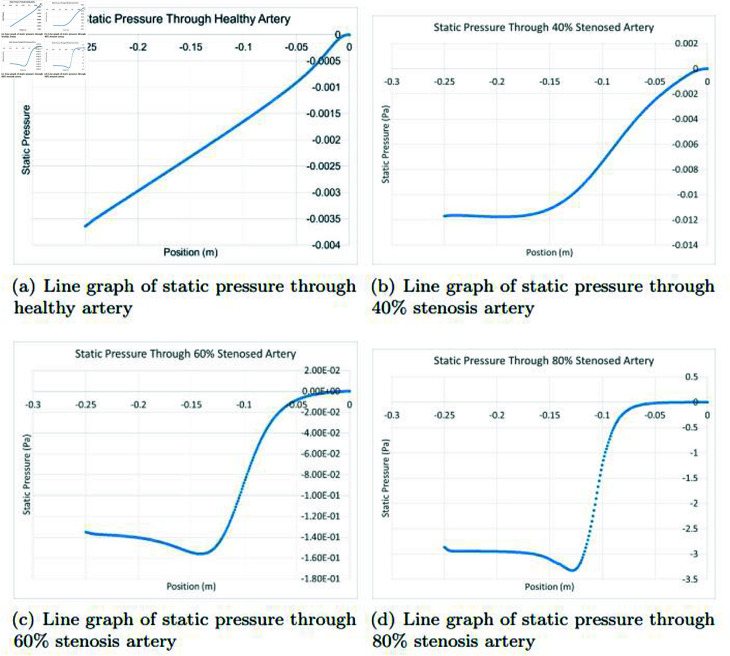
Representation of line graphs for static pressure through healthy, 40*%*, 60*%* and 80*%* stenosis artery.

In the Fig 12(a), in a healthy artery, the representation of static pressure typically reveals a linear graph that illustrates the relationship between the position along the artery and the static pressure. This linear relationship is primarily due to the principles of fluid dynamics. As blood flows through a healthy artery, the pressure remains relatively constant along the artery’s length. The arterial system is designed to maintain a consistent pressure gradient, ensuring that blood is efficiently distributed to all parts of the body. This characteristic is achieved through the elasticity and compliance of the arterial walls, which help absorb the pulsatile nature of the blood pumped by the heart. As a result, the static pressure remains fairly uniform, leading to the linear graph. In the Fig 12(b), in a 40*%* occluded artery, the representation of static pressure typically shows a curved graph rather than a linear one when illustrating the relationship between position and static pressure. This deviation from linearity is mainly a consequence of the significant blockage within the artery. The presence of a 40*%* blockage disrupts the natural, consistent flow of blood and the maintenance of a uniform static pressure gradient. As blood encounters the blockage, there’s an increase in pressure upstream of the occluded region due to the resistance posed by the narrowing of the artery. Conversely, the pressure downstream may decrease as a result of the reduced flow of blood through the partially blocked area. This alteration in pressure distribution leads to the curved graph, highlighting the impact of arterial blockages on static pressure profiles. It underscores the importance of understanding these pressure changes, as deviations from the norm can indicate potential issues in blood flow and pressure regulation, which may require medical attention and intervention to ensure adequate perfusion of tissues and organs. In the Fig 12(c), in a 60*%* occluded artery, the representation of static pressure often shows a curved graph, not a linear one. This curvature is primarily due to the significant blockage within the artery, which disrupts the natural flow of blood and the maintenance of a uniform static pressure gradient. The blockage causes an increase in pressure upstream and a decrease downstream, resulting in non-linearity. This deviation from a linear profile highlights the substantial impact of severe arterial blockages on static pressure patterns, indicating potential issues in blood flow and pressure regulation that may necessitate medical intervention to ensure adequate tissue perfusion. In the Fig 12(d), in an 80*%* occluded artery, the static pressure graph exhibits a more pronounced curve compared to healthier arteries or those with lower blockages like 40*%* and 60*%*. This substantial curvature results from the severe blockage disrupting blood flow and pressure regulation, causing a significant increase in pressure upstream and a decrease downstream of the occlusion. The non-linearity underscores the severe impact of extensive arterial blockages on static pressure profiles, necessitating urgent medical attention to ensure sufficient tissue perfusion.

### Graphically discussion of line graph for dynamic pressure

**Fig 13:**
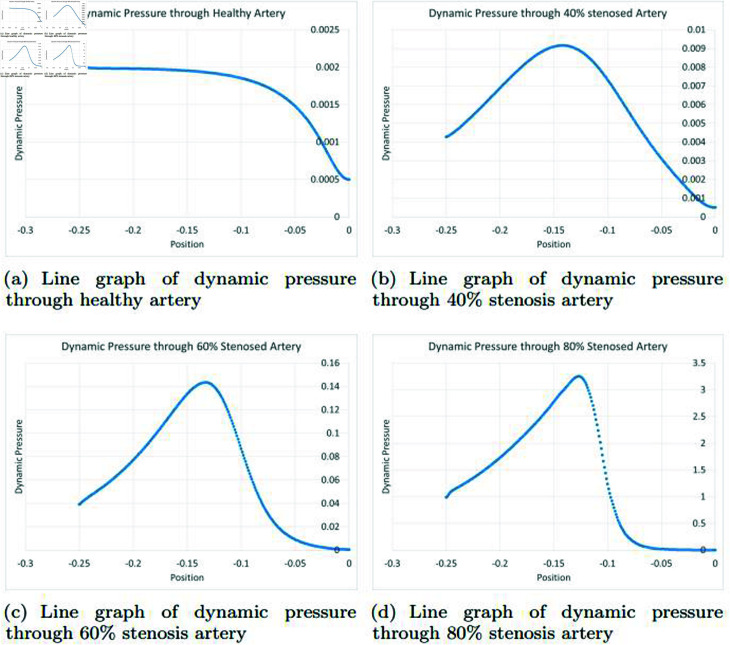
Representation of line graphs for dynamic pressure through healthy, 40*%*, 60*%* and 80*%* stenosis artery.

In the Fig 13(a), in a healthy artery, the representation of dynamic pressure typically demonstrates a graph that illustrates the relationship between position along the artery and dynamic pressure. What distinguishes this graph is the consistency of dynamic pressure as the position changes. The constant dynamic pressure along the artery is largely due to the well-maintained laminar flow of blood within the healthy vessel. In a smoothly operating artery, the blood flows in well-organized layers, and the dynamic pressure remains relatively constant, despite the changes in position. However, as the position along the artery changes further downstream, the dynamic pressure may begin to exhibit a downward trend. This decline in dynamic pressure is often associated with factors such as the expansion of the artery, reduced resistance as it approaches arterioles, or the overall damping effect that the arterial system provides to maintain a consistent flow. This downward movement signifies that the dynamic pressure reduces as the blood circulates through smaller branches and capillaries, leading to a decrease in pressure to facilitate efficient exchange of oxygen and nutrients with surrounding tissues. In the Fig 13(b), in a 40*%* occluded artery, the representation of dynamic pressure typically reveals a graph illustrating the relationship between position along the artery and dynamic pressure. What distinguishes this graph is the initial increase in dynamic pressure as the position changes within the artery. This increase at the beginning is primarily attributed to the presence of the arterial blockage. The 40*%* blockage creates resistance to blood flow, leading to higher dynamic pressure as blood encounters the narrowing of the artery. The increased dynamic pressure is a response to the heightened effort required for the blood to move past the obstruction. However, as one moves further downstream along the artery, the dynamic pressure may begin to exhibit a downward trend. This reduction in dynamic pressure is related to the adaptation of the blood flow to the blockage’s presence. Downstream, as the blood bypasses the narrowed region, it regains a more laminar and organized flow pattern. The gradual decrease in dynamic pressure reflects the restoration of smoother blood flow, with less resistance and turbulence as the artery widens or approaches smaller vessels. The dynamic pressure pattern in a 40*%* occluded artery illustrates the complex interaction between the vascular system and the impediment created by the blockage. The initial increase represents the struggle against resistance, while the subsequent decrease signifies the artery’s effort to restore more efficient blood flow. In the Fig 13(c), in a 60*%* occluded artery, the dynamic pressure graph shows an initial increase in pressure due to the substantial blockage’s resistance. However, as one moves downstream, the dynamic pressure decreases, reflecting the artery’s adaptation to the blockage, with smoother blood flow in wider sections. This complex pattern underscores the challenge the circulatory system faces in maintaining efficient blood flow in the presence of severe blockages. In the Fig 13(d), in an 80*%* occluded artery, the dynamic pressure graph initially shows an increase due to the severe blockage’s resistance. However, as one moves downstream, the dynamic pressure decreases, reflecting the artery’s adaptation to the blockage, with a return to smoother blood flow in wider sections. This dynamic pressure pattern highlights the circulatory system’s efforts to maintain efficient blood flow despite the challenges of significant arterial blockages.

### Graphically discussion of line graph for pressure coefficient

**Fig 14:**
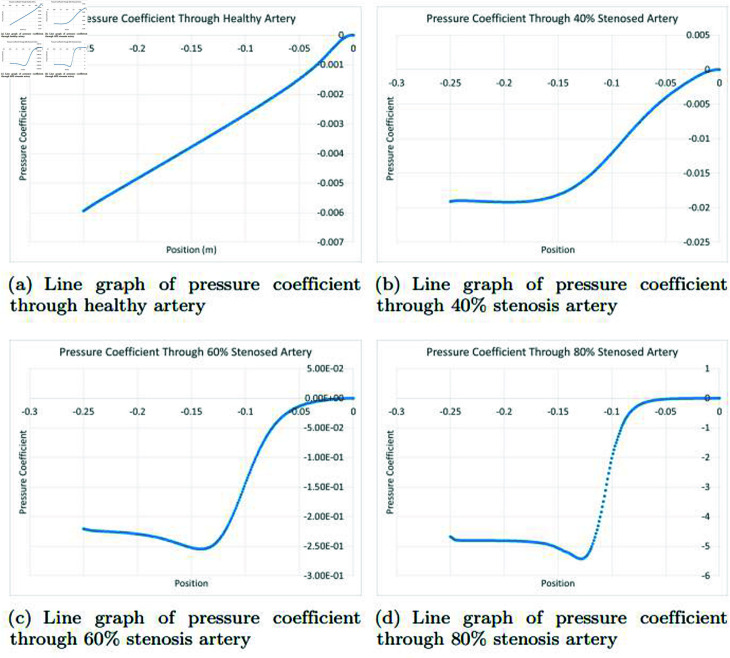
Representation of line graphs for pressure coefficient through healthy, 40*%*, 60*%* and 80*%* stenosis artery.

In the Fig 14(a), in a healthy artery, the representation of pressure coefficient typically demonstrates a linear graph when illustrating the relationship between position along the artery and the pressure coefficient. This linearity is primarily due to the well-maintained laminar flow of blood within the healthy vessel. The pressure coefficient measures the pressure relative to the static pressure in the flow, and in a smoothly operating artery, this coefficient remains relatively consistent along the artery’s length. The arterial system is designed to maintain a steady pressure gradient, which is essential for ensuring that blood is efficiently distributed to all parts of the body. This characteristic is achieved through the elasticity and compliance of the arterial walls, which help absorb the pulsatile nature of the blood pumped by the heart. The resulting linear pressure coefficient graph reflects the steady and controlled nature of blood flow within a healthy artery. However, it’s important to note that in certain pathological conditions or with the presence of stenosis, plaques, or vascular issues, this linear pressure profile may be disrupted, indicating potential problems with blood flow and pressure regulation that may require medical intervention. In the Fig 14(b), in a 40*%* occluded artery, the pressure coefficient graph typically displays a non-linear pattern. Initially, the pressure coefficient remains relatively constant during diastole, when the heart chambers relax and fill with blood. However, during systole, the phase when the heart contracts to eject blood into the arteries, the pressure coefficient increases. This is due to the additional pressure required to push blood through the narrowed artery. The non-linear pattern reflects the interaction between the arterial blockage and the pulsatile nature of blood flow during the cardiac cycle, emphasizing the need to overcome resistance to ensure effective blood distribution. In the Fig 14(c), in a 60*%* occluded artery, the pressure coefficient graph typically exhibits a non-linear pattern. Initially, during diastole, when the heart’s chambers relax and fill with blood, the pressure coefficients remain relatively constant. However, during systole, the phase when the heart contracts to eject blood into the arteries, there’s an initial decrease in pressure coefficients. This is due to the resistance posed by the 60*%* blockage, hindering efficient blood ejection. The decrease reflects the effort required to overcome this resistance. Subsequently, the pressure coefficients increase, reflecting the surge in pressure generated by the heart’s contraction to propel blood through the narrowed artery. This non-linear pattern highlights the complex interaction between the blockage and the pulsatile cardiac cycle in adapting to ensure effective blood distribution while managing the challenges introduced by the arterial narrowing. In the Fig 14(d), in an 80*%* occluded artery, the pressure coefficient graph typically displays a non-linear pattern. Initially, during diastole, when the heart’s chambers relax and fill with blood, the pressure coefficients remain relatively constant. However, as the heart transitions to systole, there’s an initial decrease in pressure coefficients due to the substantial resistance posed by the 80*%* arterial blockage. This reflects the effort needed to overcome the resistance. Subsequently, the pressure coefficients increase, reflecting the surge in pressure generated by the heart’s contraction to propel blood through the narrowed artery. This non-linear pattern highlights the complex interplay between the blockage and the pulsatile cardiac cycle in adapting to ensure effective blood distribution while managing the challenges introduced by the arterial narrowing.

### Graphically discussion of line graph for cell Reynolds number

**Fig 15:**
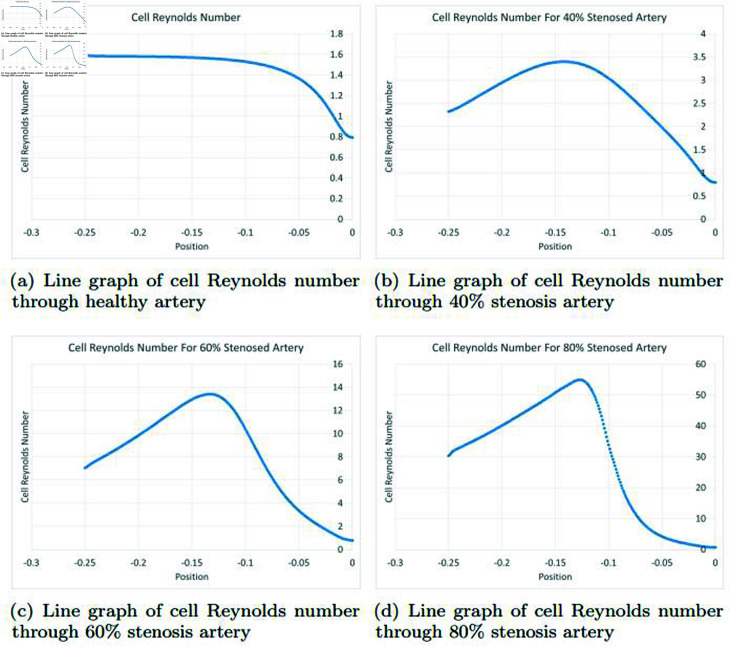
Representation of line graphs for cell Reynolds number through healthy, 40*%*, 60*%* and 80*%* stenosis artery.

The Reynolds number is a dimensionless quantity that characterizes the flow of a fluid, such as blood in arteries. It is defined as the ratio of inertial forces to viscous forces and is a critical parameter in determining the type of flow within a conduit. In the context of arterial health, the Reynolds number plays a crucial role in understanding the flow dynamics and potential implications for vascular health. In the Fig 15(a), the Reynolds number provides valuable insights into the flow dynamics within arteries. A gradual increase from  − 0 . 25 to 0 . 8 in the line graph indicates a transition from laminar to turbulent flow, with implications for arterial health. While some turbulence is normal and tolerable, excessive turbulence, as indicated by a high Reynolds number, may contribute to conditions like atherosclerosis, emphasizing the importance of maintaining a balance for optimal vascular function. In the Fig 15(b), in a scenario where there is a 40*%* stenosis (narrowing) in an artery, the Reynolds number continues to be a critical factor in understanding the flow dynamics within the vessel. The Reynolds number is influenced by factors such as blood velocity, density, viscosity, and the diameter of the vessel. In the context of a stenotic artery, the impact of the stenosis on the Reynolds number and flow patterns becomes particularly relevant. The initial value of  − 0 . 25 in the line graph suggests that, at the onset, the flow may be characterized by laminar conditions. However, the subsequent large curve and gradual increase in Reynolds number, reaching 0 . 5, indicate a notable shift in flow dynamics. This change can be attributed to the presence of the 40*%* stenosis in the artery. As the artery undergoes stenosis, the cross-sectional area available for blood flow is reduced. This reduction in diameter can lead to an increase in blood velocity through the narrowed segment. According to the Hagen–Poiseuille equation, which governs laminar flow through a cylindrical pipe, the flow rate is directly proportional to the fourth power of the radius. Therefore, even a moderate reduction in diameter can result in a significant increase in velocity and, subsequently, the Reynolds number. The larger curve in the graph and the gradual rise in Reynolds number indicate a transition toward turbulent flow. In the context of a stenotic artery, turbulent flow can exacerbate the impact of the stenosis on the vessel walls. The irregular and chaotic nature of turbulent flow can lead to heightened shear stress on the arterial walls, potentially contributing to further damage and inflammation. In the Fig 15(c), in a 60*%* stenosis artery, the Reynolds number is significantly impacted, as reflected in the line graph’s prominent curve from  − 0 . 25 to 0 . 2. The substantial narrowing of the artery increases blood velocity, leading to a transition from laminar to turbulent flow. This turbulence elevates shear stress on arterial walls, potentially exacerbating damage and inflammation. The observed graph suggests a heightened risk of vascular complications due to the pronounced impact of the stenosis on flow dynamics. In the Fig 15(d), with an 80*%* stenosis in the artery, the Reynolds number experiences a significant impact, evident in the line graph’s substantial curve from  − 0 . 25 to 0. The severe narrowing intensifies blood velocity, prompting a shift from laminar to turbulent flow. This turbulence substantially increases shear stress on the arterial walls, heightening the risk of damage and inflammation. The graph underscores the pronounced influence of an 80*%* stenosis on flow dynamics, emphasizing the elevated potential for vascular complications.

### Graphically discussion of line graph for Prandtl number

**Fig 16:**
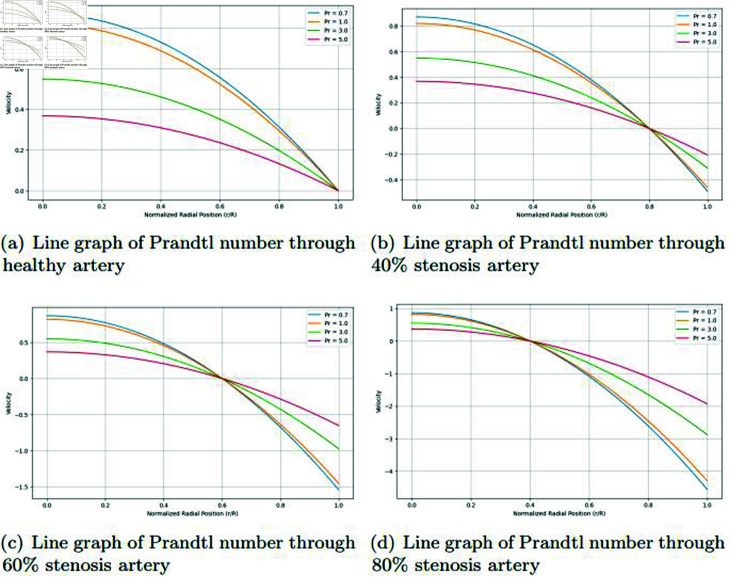
Representation of line graphs for Prandtl number through healthy, 40*%*, 60*%* and 80*%* stenosis artery.

In the Fig 16(a), in the case of a healthy artery (0% stenosis), the velocity profile follows a parabolic shape. The velocity is highest at the center of the artery and gradually decreases to zero at the artery wall (normalized radial position = 1). For different Prandtl numbers, the profiles exhibit variation. Higher Prandtl numbers (like Pr = 5) result in a steeper drop in velocity near the artery walls, while lower Prandtl numbers (Pr = 0.7) show more gradual velocity reduction. In the healthy artery, the velocity profile shows the least distortion since there is no blockage. The influence of the Prandtl number here is primarily on how quickly the velocity diminishes from the center to the artery wall. Higher Prandtl numbers indicate a faster decay of velocity near the walls, suggesting that the thermal diffusion is more dominant relative to momentum diffusion, thus affecting the boundary layer.

In the Fig 16(b), for the artery with 40% stenosis, the velocity profile becomes slightly more flattened at the center, indicating that the flow is more constrained than in a healthy artery. As the Prandtl number increases, the velocity drop near the artery walls is more pronounced, similar to the healthy case, but the overall velocity magnitude is lower due to the reduced cross-sectional area. In this case, the narrowing of the artery (40% stenosis) begins to affect the velocity profile. Blood flow is more restricted, leading to a slightly less parabolic shape compared to the healthy artery. With higher Prandtl numbers, the velocity near the artery walls decreases more sharply, showing that higher thermal diffusivity impacts the flow profile more noticeably when stenosis is present. In the Fig 16(c), for 60% stenosis, the central velocity of the artery is significantly reduced compared to the healthy and 40% stenosis cases. The velocity profiles for different Prandtl numbers are flatter in the center of the artery. The difference between high and low Prandtl numbers becomes more pronounced, with higher Prandtl numbers showing steeper velocity reductions near the walls. At 60% stenosis, the restriction of blood flow is quite severe. The velocity profile flattens more compared to earlier cases, indicating a diminished ability to sustain high flow velocities. Higher Prandtl numbers continue to cause a faster drop in velocity near the artery walls, suggesting that as thermal diffusivity becomes more significant, it further reduces the momentum transfer to the walls, particularly in more stenotic arteries. In the Fig 16(d), in the case of 80% stenosis, the velocity profile becomes almost flat near the center, and the overall velocity is much lower than in previous cases. The effect of the Prandtl number is now most evident, as higher Prandtl numbers cause extremely steep velocity reductions near the walls. The velocity for the highest Prandtl number (Pr = 5) drops almost instantaneously near the walls compared to lower Prandtl numbers. In this highly stenotic case, the blood flow is severely restricted, leading to a drastically reduced velocity profile. The influence of the Prandtl number is most significant in this case, particularly near the artery walls, where higher Prandtl numbers lead to a sharper velocity drop. This suggests that as the artery becomes more occluded, the balance between thermal diffusivity and momentum diffusion becomes more critical in shaping the velocity profile. High Prandtl numbers mean that thermal diffusion dominates, leading to more rapid velocity changes near the artery walls.

### Graphically discussion of line graph for Brownian motion parameter

**Fig 17:**
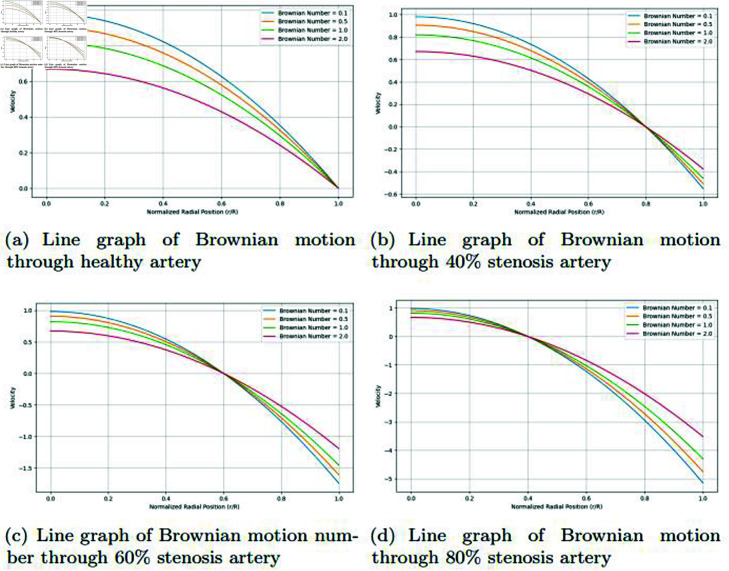
Representation of line graphs for Brownian motion through healthy, 40*%*, 60*%* and 80*%* stenosis artery.

In the Fig 17(a), for the healthy artery, the velocity profile follows the typical parabolic shape. The highest velocity is at the center (r = 0), and the velocity decreases gradually toward the artery wall (r = R). As the Brownian motion number increases (from 0.1 to 2.0), the velocity near the artery walls drops more sharply. However, the center velocity remains relatively unaffected by the Brownian motion. In a healthy artery, the flow remains relatively unimpeded, so the velocity profile maintains its classic parabolic form. The effect of Brownian motion is primarily felt near the artery walls where particle diffusion impacts momentum transfer. With larger Brownian motion numbers, the velocity near the artery wall decreases more rapidly because nanoparticle diffusion enhances the interaction between the fluid and the artery walls, slowing the flow in those regions. The core of the flow, where nanoparticle effects are less significant, remains fast. In the Fig 17(b), in the case of 40% stenosis, the artery’s narrowing causes the velocity profile to flatten slightly compared to the healthy artery, though it still maintains a parabolic shape. The effect of increasing the Brownian motion number is more pronounced here. As the Brownian motion number increases, the velocity near the walls shows a sharper decline, and the overall velocity profile starts to compress. With a 40% stenosis, the flow is partially obstructed, resulting in a reduced velocity profile. The Brownian motion impacts become more visible as the artery is narrower, meaning nanoparticles diffuse more efficiently in the smaller cross-sectional area. Higher Brownian motion numbers cause a significant reduction in velocity near the walls, emphasizing the role of particle diffusion in impeding flow near the boundaries. The central velocity, however, is still relatively high compared to more stenotic cases. In the Fig 17(c), at 60% stenosis, the artery’s narrowing causes the velocity profile to become much flatter. The central velocity is significantly lower compared to the healthy and 40% stenosis cases. The Brownian motion number’s impact is clear: as it increases, the velocity near the artery walls drops off steeply, with the entire velocity profile becoming much more compressed. In a 60% stenotic artery, blood flow is highly constrained, and the velocity profile is noticeably altered. Brownian motion enhances the diffusion of nanoparticles, further reducing the velocity near the artery walls and limiting flow across the cross-section. Higher Brownian motion numbers have a more substantial effect on the entire profile in this stenotic case, leading to a sharper velocity reduction near the walls and further flattening in the center. The flow becomes much slower and more uniform due to the severe narrowing. In the Fig 17(d), with 80% stenosis, the artery is severely constricted, and the velocity profile is nearly flat across the entire artery, showing minimal variation from the center to the walls. As the Brownian motion number increases, the velocity near the walls drops significantly, with the velocity profile becoming nearly uniform at low values. The central velocity is drastically reduced, and the velocity drop near the walls becomes almost instantaneous for higher Brownian motion numbers. In the case of severe stenosis (80%), the blood flow is extremely restricted, and the velocity profile becomes almost completely flat. Brownian motion plays a dominant role in the velocity near the walls, effectively stalling the flow as nanoparticles diffuse rapidly. For higher Brownian motion numbers, the flow near the artery walls becomes nearly stagnant, further reducing the already minimal flow due to the severe stenosis. The velocity across the artery is uniformly low, and the overall impact of Brownian motion is magnified due to the high degree of constriction.

### Advantages of obtained results

The obtained solutions offer several key advantages:

- **Enhanced accuracy in simulation:** By employing the non-Newtonian Williamson fluid model and advanced numerical methods, the solutions provide a more precise representation of blood flow dynamics in stenosed arteries. This accuracy is crucial for capturing the complex shear-thinning behavior of blood and its interaction with arterial stenoses.
- **Detailed insight into hemodynamics:** The solutions deliver comprehensive information on how different stenosis formations affect blood flow, pressure distribution, and energy loss. This detailed insight allows for a better understanding of the hemodynamic consequences of stenosis, which is valuable for clinical assessments and treatment planning.
- **Improved diagnostic and treatment strategies:** The study’s findings can lead to more accurate diagnostic tools and tailored treatment approaches. By identifying specific changes in flow patterns and energy loss associated with various stenosis types, healthcare providers can develop more effective strategies for managing arterial stenosis.
- **Refinement of computational techniques:** The use of refined mesh generation and the finite volume method enhances the precision of the simulations, ensuring reliable results. This methodological rigor can serve as a benchmark for future studies and applications in computational fluid dynamics. Overall, these advantages contribute to a deeper understanding of arterial stenosis and support advancements in both clinical and computational aspects of cardiovascular health.

## Conclusions and future initiatives

The arterial blood flow problem is studied numerically by taking into account four different types of stenotic areas. For flow inside these artery segments, a non-Newtonian model of fluid flow is taken into consideration. Key findings from this investigation are listed as follows:

- In the area of the beginning of arterial stenotic areas, the velocity profile’s magnitude is greater.
- Although the problem is modeled for a laminar flow, the results clearly demonstrate that a turbulent flow phenomenon occurs around the stenotic zones. This is because turbulence is noticed in real-life applications of an artery stenosis flow problem. Streamlines illustrate the phenomenon of turbulent flow.
- In the area of the high corners of stenotic segments, the pressure profile reaches high values.
- As a result of the narrowing of the arterial cross-section, the varied time shows that the post-stenotic segment of the artery has a higher pressure than the pre-stenotic section.
- The varied time suggests that an axially symmetric profile will eventually be the norm for the flow within the arterial portion.
- Streamlines illustrate the arterial blood flow profile visualization inside many stenotic locations.
- The current study will serve as a standard by which to compare the various non-Newtonian models’ arterial blood flow issues in the future work.
- Analysis of blood flow inside stenotic regions provides the necessary information for surgical purposes, as well as the flow pattern through arterial stenotic regions and the location of the stenosis’s origin.

Future directions for this study include:

- **Exploring additional stenosis configurations:** Investigating a wider range of stenosis shapes, sizes, and severities to further understand their impact on blood flow dynamics and energy loss.
- **Incorporating patient-specific data:** Utilizing patient-specific imaging data to create personalized models, which can provide more accurate predictions and enhance the relevance of the findings for individual clinical cases.
- **Extending to complex fluid models:** Applying more complex non-Newtonian fluid models or multi-phase flow simulations to capture additional physiological phenomena and improve model fidelity.
- **Integrating with clinical tools:** Developing and integrating these findings into clinical diagnostic tools and software for real-time analysis and decision-making in medical practice.
- **Longitudinal studies:** Conducting longitudinal studies to assess how changes in stenosis over time affect blood flow and energy loss, potentially linking these dynamics to disease progression and treatment outcomes.
- **Experimental validation:** Performing experimental studies or collaborations with medical imaging facilities to validate the simulation results and ensure their applicability to real-world clinical scenarios.
